# Characterization of the Key Aroma Compounds in Different Aroma Types of Chinese Yellow Tea

**DOI:** 10.3390/foods12010027

**Published:** 2022-12-21

**Authors:** Xin Hong, Chao Wang, Ronggang Jiang, Tengfei Hu, Xuexue Zheng, Jianan Huang, Zhonghua Liu, Qin Li

**Affiliations:** 1 Key Laboratory of Tea Science of Ministry of Education, Hunan Agricultural University, Changsha 410128, China; 2 Collaborative Innovation Centre of Utilization of Functional Ingredients from Botanicals, Hunan Agricultural University, Changsha 410128, China; 3 National Research Center of Engineering Technology for Utilization of Functional Ingredients from Botanicals, Hunan Agricultural University, Changsha 410128, China

**Keywords:** yellow tea, aroma types, key aroma compounds, sensory attributes, multivariate analysis

## Abstract

Yellow tea is one of the six major tea categories in China. The floral fragrance type, high-fired fragrance type, fresh fragrance type, and corn-like fragrance type were the main aroma types of yellow tea screened by QDA. A total of 103 volatiles were identified in yellow teas by HS-SPME/GC-MS analysis. Using multivariate analysis and OAVs, forty-seven aroma compounds were identified as key aroma compounds for the formation of different aroma types of yellow teas. Among them, 8, 14, 7, and 18 key aroma compounds played an important role in the formation of aroma characteristics of floral fragrance, high-fired fragrance, fresh fragrance, and corn-like fragrance types of yellow teas, respectively. Furthermore, PLS analysis revealed that 12 aroma compounds were the key contributors to the ‘floral and fruity’ and ‘sweet’ attributes, five aroma compounds contributed to the ‘roasted’ attribute, and four aroma compounds related to the ‘fresh’ and ‘grassy’ attributes. This study provides new insights into the aroma characteristics formation of different aroma types of yellow teas and will provide a valuable theoretical basis for improving the flavor quality of yellow tea during the manufacturing process.

## 1. Introduction

Tea is a kind of widely consumed beverage ranking second only to water around the world [1]. Yellow tea is one of the six major tea categories in China, which is famous for its unique appearance and flavors, such as yellow dry tea, yellow infusion, and yellow brewed leaves that have an elegant fragrance and smooth taste. Meanwhile, yellow tea also possesses several healthy benefits for humans, such as anti-cancer, anti-bacterial, antioxidant, and gastrointestinal protecting effects [2,3,4]. The main manufacturing process of yellow tea are fixing, rolling, sealed yellowing, and drying. Among them, sealed yellowing is the key processing stage that contributes to the typical flavor characteristic formation of yellow tea [2]. Although yellow tea is increasingly deeply loved by consumers, its key aroma compounds that responsible for the special aroma formation are still unknown. It is difficult to establish flavor directed processing technology for yellow tea. Therefore, the study of yellow tea, particularly, research on its key aroma compounds for this special aroma, has important value for improving the flavor quality of yellow tea during the manufacturing process.

Tea aroma is a critical criterion to evaluate the overall quality of yellow tea [4]. Many aroma components were identified in yellow tea by previous studies, but different yellow tea possesses a wide range of key aroma compounds. This might result from the different raw materials, processes, and parameters of different yellow teas. The detailed information is summarized in Table S1. Our previous study indicated that different levels of tenderness of tea leaves will form the different characteristic aromas and key aroma compounds in yellow tea. Phenylacetaldehyde was identified as the key aroma compound in the bud-type yellow tea, due to its special fresh aroma; Ethyl hexanoate, benzyl alcohol, geraniol, phenethyl alcohol, citral, neral, and myrcene were identified as major contributors in bud-leaf yellow tea due to their flowery, fruity and sweet aroma notes; 2-pyrrole formaldehyde, 3-ethyl-2,5-dimethylpyrazine, 2-ethyl-5-methylpyrazine, and 2,3-diethyl-5-methylpyrazine are the key aroma compounds in multi-leaf yellow tea due to their roasted aroma notes [5]. Another study also revealed that key aroma compounds, including (*E,E*)-3,5-octadien-2-one, (*Z*)-linalool oxide (furanoid), (*E*)-2-heptenal, naphthalene, geraniol, (*E*)-linalool oxide (furanoid), styrene, linalool, α-ionone, 1-octen-3-ol, (*E*)-β-ionone, and (*E,Z*)-3,5-octadien-2-one, presented great differences in the different tenderness levels of yellow tea [6]. In addition, the different manufacturing processes can significantly affect the aroma profile of yellow tea and lead to the different characteristics of aroma in yellow tea. A previous study revealed that the high temperature roasting process was essential for special flavor formation of yellow teas with strong ‘roasted’, ‘nutty’, and ‘woody’ odors, as well as weak ‘fatty’ and ‘fruity’ odors [7]. This was also the key process for unique ‘crispy-rice-like’ odor formation in yellow tea [8]. In addition, different temperatures of roasting processes could result in different aroma characteristics of yellow tea, causing odors such as ‘fresh’, ‘tender corn’, ‘ripe corn’, and so on [9]. The degree of yellowing process significantly affects the composition of the aroma profile in yellow tea [10].

In summary, it is reasonable to hypothesize that different raw materials and processing technologies will lead to different compositions of the aroma profile, as well as cause different types of characteristic aromas in yellow tea. However, up to now, there are scarcely any systematic comparative study focused on the composition of key aroma compounds in different aroma types of yellow tea. Therefore, in this study, our goals are to (a) screen out the main aroma types of yellow tea by sensory-directed flavor analysis; (b) identify the key aroma compounds responsible for characteristic aroma formation in the different aroma types of yellow tea; (c) illuminate the correlation the aroma compounds and sensory attributes. Our findings will provide important information for the key aroma compounds that contribute to the different characteristic aroma formation of yellow tea, and also provide valuable information for improving the aroma quality of yellow tea during the manufacturing process.

## 2. Materials and Methods

### 2.1. Sample Collection and Chemicals

Ninety-seven yellow tea samples were collected from the tea market all over China in 2021 (Table S2). The C7-C40 n-alkanes and ethyl decanoate (99.99%) were obtained from Sigma-Aldrich (St. Louis, MO, USA). Fifty authentic standards were purchased from J&K Chemical Ltd. (Beijing, China) (Table S3). All the solvents were of chromatography grade, and all the chemicals were of analytical reagent grade, unless otherwise stated.

### 2.2. Sensory Analysis

Sensory analysis was approved by the Hunan Agricultural University Institutional Review Board Committee (TSF-780-2021). Twelve panelists (six males and six females, aged from 25 to 55 years) were selected from the Tea Science Department in Hunan Agricultural University. All participants received written information about the study and signed informed consent forms to participate. The yellow tea samples with representative aroma characteristics were selected by sensory evaluation according to the Chinese standards ‘Methodology of Sensory Evaluation of Tea’ (GB/T 23776-2018) and submitted to the quantitative descriptive analysis (QDA) [11]. Each sample was evaluated three times. The detailed information about sensory evaluation is described in Supplement Material S1.

### 2.3. Qualitative and Quantitative Analysis

The volatile compounds were analyzed by the HS-SPME/GC-MS method according to our previous study [12]. Briefly, each sample was initially ground and homogenized. The tea powder (1 g), NaCl (0.5 g), and ethyl decanoate (10 μL, 8.64 mg/L) were introduced into a 20 mL headspace bottle and infused with 5 mL of boiling water. The vial was immediately sealed and kept at 80 °C for 10 min. A 65-μm polydimethylsiloxane/divinylbenzene (PDMS/DVB) fiber (Supelco, Bellefonte, PA, USA) was exposed to the sample for 40 min. Next, the SPME fiber was inserted into GC injection port (230 °C for 5.0 min) and subsequently analyzed. Each sample was analyzed three times.

GC conditions: An Agilent 7890B GC system coupled with an Agilent 5977A MSD mass spectrometer (Agilent, Santa Clara, CA, USA) was used. An Agilent HP-5MS capillary column (30 m × 0.25 mm i.d. × 0.25 μm film thickness) was used for separation; the carrier gas was helium (purity > 99.999%) with a constant flow rate of 1 mL/min. The injection mode was set to splitless. The oven temperature was initially held at 50 °C for 3 min, raised to 170 °C at a rate of 3 °C/min, raised to 190 °C at a rate of 5 °C/min, then raised to 250 °C at a rate of 15 °C/min, and finally held at temperature for 2 min. MS conditions: EI ionization energy, ion source temperature, quadrupole temperature, and mass scanning range were set to 70 eV, 230 °C, 150 °C, and 35–400 atomic mass units (amu), respectively. Retention indices (RIs), authentic standards, and mass spectra matching in the standard NIST17 library were used for identification. RIs were calculated after analyzing C7-C40 n-alkane series under the same chromatographic conditions. Moreover, some commercially available standards were applied to verify the analysis results under the same conditions. The volatile compounds with available standards were quantitated according to our previous study. For the volatile compounds without the available standards, the quantitation was carried out using the standard that had the same carbon atom or a similar functional structure [12].

### 2.4. Odor Activity Value

Odor activity values (OAVs) are frequently applied to evaluate the contributions of aroma compounds. The compound with OAV > 1 was considered as key a aroma-active compound, which significantly contributed to the formation of aroma characteristics. The OAV was calculated as the ratio of the dividing the calculated concentration of each aroma compound to its odor threshold (OT) in water. The aroma characteristics and OTs were taken from previous literature [6,7,13,14,15,16,17,18,19,20,21] and online databases (FEMA, https://www.femaflavor.org/ and TGSC, http://www.thegoodscentscompany.com/search2.html (accessed on 1 March 2022)).

### 2.5. Multivariate Analysis

The data were preprocessed by mean centering and scaling prior to analysis. Principal component analysis (PCA), hierarchical cluster analysis (HCA), supervised orthonormal partial least-squares discriminant analysis (OPLS-DA), and partial least-squares analysis (PLS) were performed with SIMCA-P+ (Version 14.0, Umetrics, Umea, Sweden). The aroma compounds were used as the X variables and the aroma sensory attributes as the Y variables for PLS analysis. All data were presented as the mean values ± SD. Significant differences between groups were declared significant at *p* < 0.05.

## 3. Results and Discussion

### 3.1. Different Aroma Type of Yellow Tea

**Figure 1:**
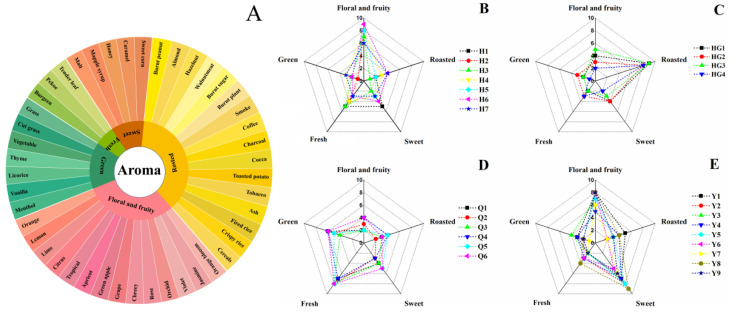
Flavor wheel of yellow tea (**A**) and radar of sensory aroma notes profile among the four aroma types of yellow tea: (**B**) ‘floral fragrance’ type. (**C**): ‘high-fired fragrance’ type. (**D**): ‘fresh fragrance’ type. (**E**): ‘corn-like fragrance’ type.

In this study, 26 yellow tea samples with typical aroma characteristics were selected from 97 yellow tea samples for QDA (Table S2). As shown in Figure 1A, a flavor wheel was established by sensory evaluation. The flavor wheel consisted of three tiers, one first-tier descriptor, five s-tier descriptors, and forty-six third-tier descriptors. The five s-tier descriptors have been widely used to describe the aroma characteristics of yellow tea in previous studies, including ‘floral and fruity’, ‘roasted’, ‘sweet’, ‘fresh’, and ‘green’ [7,8].

According to the second-tier descriptors and their sensory intensities, the aroma characteristics of 26 yellow tea samples could be divided into four aroma types, namely floral fragrance (H1-H7), high-fired fragrance (HG1-HG4), fresh fragrance (Q1-Q6), and corn-like fragrance (Y1-Y9). The floral fragrance type of yellow tea had a predominantly ‘floral and fruity’ attribute, whereas the other attributes were only weakly present (Figure 1B). The high-fired fragrance type of yellow tea mainly featured high intensity in the ‘roasted’ attribute, while the ‘floral and fruity’ attribute was moderate (Figure 1C). The fresh fragrance type of yellow tea had a strong ‘fresh’ attribute, while the ‘green’ and ‘sweet’ attributes were moderate (Figure 1D). The corn-like fragrance type of yellow tea was marked by strong ‘sweet’ and ‘floral and fruity’ attributes, while the ‘roasted’ attribute was moderate (Figure 1E).

### 3.2. Volatile Profile in Different Aroma Types of Yellow Tea

The volatile profiles of different aroma types of yellow teas were analyzed by HS-SPME/GC-MS with external standard method (Table S3). A total of 103 volatile compounds were identified and quantified in the different aroma types of yellow teas (Table 1), and 101, 91, 90, and 97 volatile compounds were identified in the floral fragrance, high-fired fragrance, fresh fragrance, and corn-like fragrance types of yellow teas, respectively. These identified volatile compounds belong to seven categories, including 16 alcohols, 2 phenols, 13 nitrogenous compounds, 10 aldehydes, 33 hydrocarbons, 13 ketones, and 16 esters. As shown in Figure S1A, alcohols (33.48%) and aldehydes (21.90%) were the predominant categories in the floral fragrance type of yellow tea, and (*Z*)-linalool oxide (furanoid), (*E*)-linalool oxide (furanoid), linalool oxide (pyranoid), hotrienol, (*Z*)-citral, and citral were the most abundant volatiles compared with the other aroma types of yellow tea (Figure S1B). Indeed, the alcohols and aldehydes have been reported to provide ‘floral’ and ‘fruity’ attributes, which have a good coordinating effect on the aroma profiles of tea products [6,22]. Nitrogenous compounds (60.10%) and hydrocarbons (17.27%) were the main categories in the high-fired fragrance type of yellow teas (Figure S1A). Among them, 1-ethyl-1H-pyrrole, 2,4-dimethyl-3-ethylpyrrole, 2,5-dimethyl-3-ethylpyrazine, 2-methyl-5-ethylpyrazine, methyl anthranilate, and p-xylene were the most abundant volatile compounds compared with the other aroma types of yellow teas (Figure S1B). These pyrroles, pyrazines, and their derivatives are usually generated from the Maillard reaction during roasting and baking treatments, which are thought to contribute to the formation of the ‘roasted’ aroma [1,7]. Esters (45.06%) and alcohols (28.03%) were the dominant categories in the fresh fragrance type of yellow tea (Figure S1A). Among them, ethyl hexanoate, ethyl nonanoate, and (E)-3-hexen-1-ol were the most abundant chemical structures, compared with the other aroma types of yellow tea (Figure S1B). Esters have been reported to play an important role in the fresh aroma characteristics formation of yellow tea [6]. Hydrocarbons (21.68%) and ketones (11.62%) were the dominant categories in the corn-like fragrance type of yellow tea (Figure S1A). Compared with the other aroma types, styrene, (*E*)-β-ocimene, β-cedrene, toluene, mesityl oxide, 2,3-octanedione, and β-ionone were the most abundant in the corn-like fragrance type (Figure S1B). Hydrocarbons and ketones with ‘sweet’, ‘floral’, and ‘fruity’ aroma notes, which are found to dominate the volatile compounds of black tea and are considered to provide a unique aroma for black tea [23,24]. The results showed that there were significant differences in the composition of volatile compounds of yellow teas with different aroma types. 

**Figure 2:**
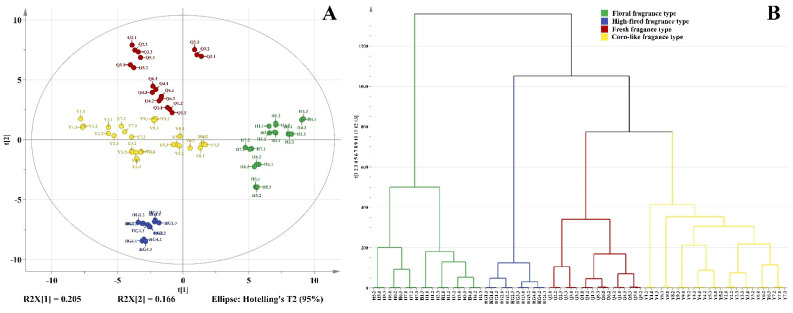
PCA (**A**) and HCA (**B**) of the different aroma types of yellow teas.

To investigate the similarities and differences of volatile compounds among the four aroma types of yellow teas, PCA and HCA were performed based on the quantitative results of the identified volatile compounds. The results of PCA (the total variance of model was 89%) (Figure 2A) and HCA (Figure 2B) showed that the yellow teas could be divided into four groups according to their aroma types, which indicated that there were significant differences in the volatile profile among the four aroma types of yellow teas. Based on these results, six OPLS-DA models were established to investigate the discriminatory volatile compounds (Figure S2). Among the four types of yellow tea, 75 discriminative volatile compounds, which might play an important role in the formation of different aroma characteristics and contribute to the distinction of these four aroma types of yellow teas (Figure S3), were identified.

### 3.3. Key Aroma Compounds in Different Aroma Types of Yellow Tea

It is well known that the characteristic aroma of yellow tea is formed by a series of volatile compounds with a certain composition and proportion. The characteristic aroma mainly depends on the concentrations of aroma compounds and their OAVs. OAV is usually used to evaluate the contribution of the aroma compound to the odor of tea [13]. Therefore, the aroma attributes and OAVs of all the identified volatile compounds were calculated and listed in Table S4. Sixty-one volatile compounds were identified with OAV > 1 in at least one aroma type of yellow tea. Twenty-four volatile compounds with OAVs > 1 were common in all yellow tea samples, and 12 (linalool, phenethyl alcohol, 1-octen-3-ol, α-ionone, β-ionone, dehydro-β-ionone, (*E,E*)-2,4-heptadienal, phenyl acetaldehyde, methyl anthranilate, ethyl hexanoate, myrcene, and α-muurolene) had OAVs > 10. Most of aroma compounds with high OAVs (OAV > 10) possess ‘floral’, ‘fruity’, ‘citrus’, ‘green’, and ‘fatty’ odors, which endow yellow tea with clean and pure scents and play a major role in the formation of a variety of aroma types of yellow teas [6].

**Figure 3:**
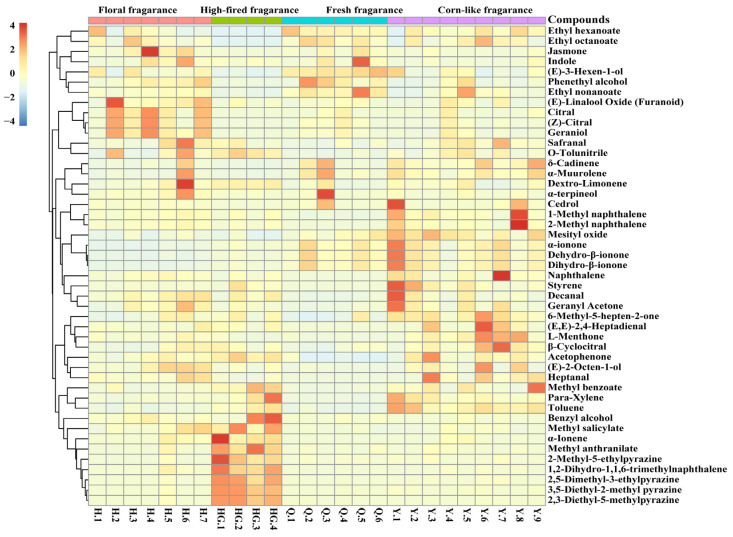
Heatmap of the discriminatory aroma compounds in the different aroma types of yellow teas.

Combined with the results of OPLS-DA, a total of 47 discriminative aroma compounds were identified in the four aroma types of yellow tea based on the three conditions: the values of predictive component variable importance in the projection (VIP) ≥ 1.0, *p* ≤ 0.05 and OAV > 1 (Figure 3). Among these discriminative aroma compounds, eight, fourteen, seven, and eighteen aroma compounds with the highest levels in the floral fragrance, high-fired fragrance, fresh fragrance, and corn-like fragrance types of yellow teas, respectively. High contents of these discriminative aroma compounds might play an important role in the formation of different aroma characteristics of yellow tea. Therefore, these discriminative aroma compounds were recognized as key aroma compounds in different aroma types of yellow tea.

In the floral fragrance types of yellow tea, (*Z*)-citral, citral, jasmone, (*E*)-linalool oxide (furanoid), decanal, geraniol, (*E*)-2-octen-1-ol, and safranal were identified as the key aroma compounds. Among them, (*Z*)-citral and citral with ‘citrus’ odor have been reported to be responsible for the formation of strongly fruity fragrance in lemon basil and hops [25,26]. €-Linalool oxide (furanoid) possesses a ‘floral’ odor and has been widely detected in yellow tea, green tea, black tea, and oolong tea. It has been considered as the important compound that contributes to ‘floral’ aroma formation in tea products [1,6,8,27,28]. In addition, as a vital odoriferous compound of honey, safranal has been regarded as an important aroma-active compound because of its ‘ripe fruit’ and ‘honey’ odor [6,14,16,29]. (*E*)-2-Octen-1-ol emits a ‘grassy’ odor and has been considered as an aroma-active compound responsible for the ‘green’ aroma characteristics formation of asam sunti [30].

In high-fired fragrance type of yellow tea, 3,5-diethyl-2-methylpyrazine, 2-methyl-5-ethylpyrazine, 2,3-diethyl-5-methylpyrazine, 2,5-dimethyl-3-ethylpyrazine, o-tolunitrile, dextro-limonene, α-ionene, acetophenone, methyl anthranilate, benzyl alcohol, methyl benzoate, 1,2-dihydro-1,1,6-trimethylnaphthalene, methyl salicylate, and para-xylene were identified as the key aroma compounds. Pyrazines, usually possess a ‘roasted’ attribute generated by heat treatment and exist abundantly in a large number of cooked, roasted, and toasted foods. For example, 3,5-diethyl-2-methylpyrazine, 2,3-diethyl-5-methylpyrazine, 2-methyl-5-ethylpyrazine, and 2,5-dimethyl-3-ethylpyrazine produce a ‘roasted hazelnut’ note, and have are considered as important contributors for the ‘roasted’ odor formation of yellow tea, green tea, coffee, and coco [7,8,9,31]. In addition, para-xylene with a ‘roasted’ attribute was identified as an aroma compound in yellow tea. This has long been considered to be a key characteristic aroma marker for the roasting process of oolong tea [7,32]. In addition, methyl anthranilate has a typical ‘grape juice’ odor that can improve the ‘floral and fruity’ aroma characteristics in many kinds of tea [6,8,12,33].

In the fresh fragrance type of yellow tea, indole, phenethyl alcohol, (*E*)-3-hexen-1-ol, α-terpineol, ethyl hexanoate, ethyl nonanoate, and ethyl octanoate were identified as the key aroma compounds. Esters are well-known aroma compounds that play an important role in the properties of beverages. Ethyl nonanoate, ethyl hexanoate, and ethyl octanoate are the representative ester compounds that can provide ‘floral’, ‘fruity’, and ‘waxy’ odors [6,34]. For example, ethyl hexanoate has been identified as an important aroma compound in oolong tea with a ‘pineapple’ odor, which contributes to the ‘sweet fruit’ aroma formation of special aroma characteristics of black tea [23,35]. Ethyl nonanoate has been considered as a potential aroma-active compound in fruits, and it also contributes to the formation of ‘rose-like’ aroma in black tea [35,36]. In addition, (*E*)-3-Hexen-1-ol has a ‘green’ note and has been reported to contribute to the formation of ‘green’, ‘grass’, and ‘fresh’ odors of black tea and oolong tea [16,23].

In corn-like fragrance of yellow tea, styrene, α-ionone, dehydro-β-ionone, dihydro-β-ionone, methyl heptenone, geranyl acetone, toluene, β-cyclocitral, mesityl oxide, naphthalene, 1-methylnaphthalene, 2-methylnaphthalene, δ-cadinene, cedrol, α-muurolene, heptanal, (*E,E*)-2,4-heptadienal, and l-menthone were identified as the key aroma compounds. Among them, styrene and toluene possessed ‘sweet balsam’ and ‘floral’ odors, which were also detected in yellow tea, black tea and are considered to play an important role in the formation of their aroma characteristics [6,8,24]. In addition, mesityl oxide presents a ‘honey’ odor and has been found in green tea and yellow tea, which contributes to the formation of a characteristic ‘sweet’ aroma [5,37]. As an unsaturated aldehyde, (*E,E*)-2,4-heptadienal is derived from the oxidation of polyunsaturated fatty acids and has ‘fatty’, ‘green’, and ‘nutty’ notes, and has been identified as a key aroma compound to form the special aroma of melons and green tea [37,38].

In addition, characteristic aromas were also impacted by the interactions among volatile compounds or volatile and non-volatile compounds [39]. Zhu et al. revealed that mixed aroma compounds with similar structures mainly present a synergistic effect and additive action, and a masking effect was found among aroma compounds with different structures in oolong tea [39]. Vicente Ferreira et al. also reported that the non-volatile higher alcohol content had an important impact on the characteristic aroma formation of wine [40,41,42].

### 3.4. Relationship between Aroma-Active Compounds and Sensory Attributes

**Figure 4:**
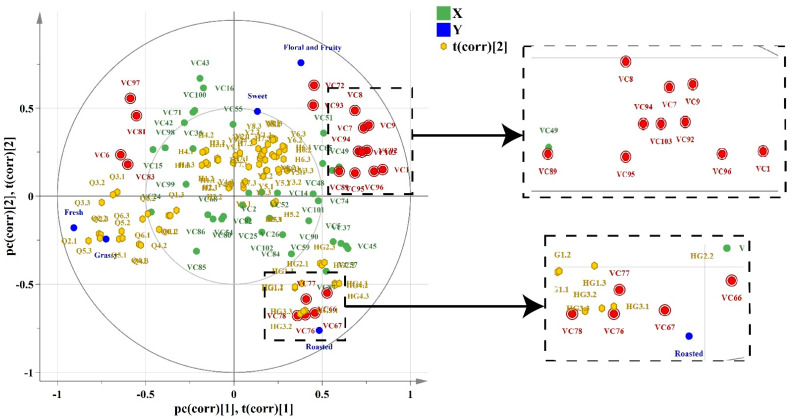
PLS plots for the tea samples, the sensory analysis, and active-aroma volatiles (OAV > 1). VC1: acetophenone; VC6: (E)-3-hexen-1-ol; VC7: 2-methylnaphthalene; VC8: naphthalene; VC9: 1-methylnaphthalene; VC66: 3,5-diethyl-2-methyl pyrazine; VC67: 2,3-diethyl-5-methylpyrazine; VC72: l-menthone; VC76: methyl anthranilate; VC77: 2,5-dimethyl-3-ethylpyrazine; VC78: 2-methyl-5-ethylpyrazine; VC81: ethyl hexanoate; VC83: ethyl nonanoate; VC89: safranal; VC92: decanal; VC93: heptanal; VC94: methyl heptanone; VC95: toluene; VC96: para-xylene; VC97: ethyl octanoate; VC103: styrene.

To explore the relationships between the aroma-active compounds (OAV > 1) and sensory attributes, a PLS model was established. Two latent variables were included in the PLS model, which represented 86.90% of X-matrix variance (aroma-active compounds) and explained 99.30% of Y-matrix variance (sensory attributes). As shown in Figure 4, the positions of the sensory attributes and aroma-active compounds of yellow tea between the two ellipses indicated that they were well explained by the PLS model. Among these samples, the floral fragrance (H1–H7) and corn-like fragrance types of yellow teas (Y1–Y9) are mainly located on the upper part of PC2. The fresh fragrance type of yellow tea samples (Q1–Q6) are mainly located in the negative region of PC1 and PC2. The high-fired fragrance type yellow tea samples (HG1–HG4) are mainly located in the positive region of PC1 and negative region of PC2. This clearly indicates that tea samples can be divided into four groups according to their aroma types.

The five attributes (‘floral and fruity’, ‘roasted’, ‘fresh’, ‘grassy’, and ‘sweet’) that significantly correlated with some aroma-active compounds were located between the inner and outer ellipses. The ‘floral and fruity’, ‘sweet’, and ‘roasted’ attributes were located on the positive dimension, and the ‘fresh’ and ‘green’ attributes were located on the negative dimension of the first PC1. The floral fragrance and corn-like fragrance types of yellow tea samples were strongly associated with ‘floral and fruity’ and ‘sweet’ attributes, the high-fired fragrance type of yellow tea was strongly associated with ‘roasted’ attribute, and the fresh fragrance type of yellow tea was strongly associated with ‘fresh’ and ‘green’ attributes. Furthermore, the ‘floral and fruity’ and ‘sweet’ attributes were positively correlated to l-menthone, heptanal, naphthalene, 2-methylnaphthalene, 1-methylnaphthalene, methyl heptenone, decanal, styrene, safranal, toluene, para-xylene, and acetophenone. A previous study suggested that the ‘floral and fruity’ attribute was related to the ‘sweet’ attribute in tea [43]. The ‘roasted’ attribute was positively correlated to methyl anthranilate, 2,3-diethyl-5-methylpyrazine, 2-methyl-5-ethylpyrazine, 2,5-dimethyl-3-ethylpyrazine, and 3,5-diethyl-2-methyl pyrazine. It is well known that the intensity of the ‘roasted’ attribute mainly depends on the content of pyrazine compounds, which has been confirmed by a previous study [1]. The ‘fresh’ and ‘green’ attributes were positively correlated with (*E*)-3-hexen-1-ol, ethyl hexanoate, ethyl nonanoate, and ethyl octanoate. Previous studies have shown that ethyl esters with low detection thresholds usually exist in large amounts in wine and play a vital role in the formation of fruity aroma characteristics [44]. Meanwhile, it has also been reported that these esters make an important contribution to the fresh aroma characteristics of yellow tea, which differs from green and black teas [6].

## 4. Conclusions

According to the difference of aroma characteristics, yellow teas were divided into four main aroma types by QDA, including the floral fragrance, high-fired fragrance, fresh fragrance, and corn-like fragrance. Among them, floral fragrance yellow tea had a predominant ‘floral and fruity’ attribute, high-fired fragrance yellow tea was mainly featured with high intensity in the ‘roasted’ attribute, fresh fragrance yellow tea had a strong ‘fresh’ attribute, and corn-like fragrance yellow tea was marked by strong ‘sweet’ and ‘floral and fruity’ attributes. In addition, a total of 103 volatile compounds were identified and quantified in yellow teas by HS-SPME/GC-MS. According to multivariate statistical analysis and OAVs, 47 volatile compounds were identified as the key aroma compounds for the formation of different aroma types of yellow tea. Among them, eight aroma compounds dominated with ‘floral and fruity’ attribute in the floral fragrance type, fourteen aroma compounds dominated with ‘roasted’ attribute in the high-fired fragrance type, seven aroma compounds dominated with ‘floral and fruity’ and ‘grassy’ attributes in the fresh fragrance type, and eighteen aroma compounds dominated with ‘floral and fruity’ and ‘sweet’ attributes in the corn-like fragrance type. Furthermore, PLS analysis revealed that the ‘floral and fruity’ and sweet attributes were positively correlated to l-menthone, heptanal, naphthalene, 2-methylnaphthalene, 1-methylnaphthalene, methyl heptenone, decanal, styrene, safranal, toluene, para-xylene, and acetophenone. The ‘roasted’ attribute was positively correlated to methyl anthranilate, 2,3-diethyl-5-methylpyrazine, 2-methyl-5-ethylpyrazine, 2,5-dimethyl-3-ethylpyrazine, and 3,5-diethyl-2-methyl pyrazine. The ‘fresh’ and ‘grassy’ attributes were positively correlated to (*E*)-3-hexen-1-ol, ethyl hexanoate, ethyl nonanoate, and ethyl octanoate. Overall, this study provides new insights into the key aroma compounds that are responsible for the characteristic aroma in different aroma types of yellow tea, and will provide an important theoretical basis for establishing flavor directed processing of yellow tea.

## Figures and Tables

**Table 1:** Identification of the volatiles in different aroma types of yellow tea.

Compounds	CAS	RI ^a^/ RI ^b^	Identification ^c^	Floral Fragrance Type	High-Fired Fragrance Type	Fresh Fragrance Type	Corn-Like Fragrance Type
				Concentration μg/L ^d^			
Alcohol compounds							
*α*-Terpineol	98-55-5	1188/1187	MS, RI, STD	225.15 ± 233.04 a	5.83 ± 2.92 b	12.9 ± 27.31 b	2.9 ± 3.76 b
(*E*)-3-Hexen-1-ol	928-97-2	852/845	MS, RI, STD	4780.39 ± 2834.72 a	95.09 ± 37.48 b	465.72 ± 172.67 b	220.41 ± 299.25 b
Linalool	78-70-6	1098/1098	MS, RI, STD	516.47 ± 267.65 a	21.68 ± 2.7 b	31.02 ± 9.65 b	25.35 ± 22.21 b
Cedrol	77-53-2	1598/1598	MS, RI, STD	90.87 ± 57.19 a	0 ± 0 b	10.44 ± 21.85 b	14.92 ± 29.72 b
Nerolidol	7212-44-4	1559/1560	MS, RI, STD	54.22 ± 82.44 a	1.35 ± 0.89 b	1.36 ± 2.03 b	0.72 ± 0.73 b
Phenethyl alcohol	60-12-8	1110/1109	MS, RI, STD	261.77 ± 202.52 a	4.77 ± 0.95 b	12.93 ± 8.27 b	7.16 ± 11.47 b
(*Z*)-Linalool oxide (Furanoid)	5989-33-3	1072/1069	MS, RI	4366.05 ± 5485.02 a	167.36 ± 39.01 b	52.29 ± 21.05 b	103.34 ± 191.67 b
4-Terpinenol	562-74-3	1179/1174	MS, RI, STD	33.63 ± 32.84 a	1.45 ± 0.48 b	1.02 ± 1.03 b	0.75 ± 0.83 b
Olivetol	500-66-3	1528/1523	MS, RI, STD	331.77 ± 180.81 a	12.28 ± 7.09 b	4.57 ± 3.69 b	5.11 ± 4.6 b
(*E*)-Linalool oxide (Furanoid)	34995-77-2	1085/1085	MS, RI	5055.04 ± 5418.54 a	153.71 ± 52.51 b	65.77 ± 32.59 b	111.81 ± 215.29 b
1-Octen-3-ol	3391-86-4	974/974	MS, RI, STD	190.28 ± 150.79 a	10.94 ± 4.92 b	8.26 ± 3.52 b	16.1 ± 14.42 b
Hotrienol	29957-43-5	1101/1102	MS, RI	2385.59 ± 2896.62 a	167.73 ± 90.58 b	23.79 ± 13.41 b	23.42 ± 24.67 b
(*E*)-2-Octen-1-ol	18409-17-1	1068/1065	MS, RI	67.6 ± 58.93 a	2.8 ± 1.33 b	0.12 ± 0.17 b	2.61 ± 3.19 b
Linalool oxide (Pyranoid)	14049-11-7	1174/1171	MS, RI	1214.45 ± 1380.18 a	10.03 ± 5.67 b	17.92 ± 14.76 b	20.89 ± 25.25 b
Geraniol	106-24-1	1251/1252	MS, RI, STD	256.28 ± 271.23 a	1.47 ± 0.2 b	2.15 ± 1.78 b	3.15 ± 6.99 b
Benzyl alcohol	100-51-6	1031/1030	MS, RI, STD	163.51 ± 217.15 a	61.32 ± 61.29 b	1.66 ± 1.81 b	1.29 ± 2.12 b
Phenols							
2,4-Di-tert-butyl phenol	96-76-4	1503/1509	MS, RI	35.76 ± 24.47 a	15.81 ± 16.34 b	2.45 ± 1.85 b	2.13 ± 2.33 b
Butylated hydroxytoluene	128-37-0	1513/1511	MS, RI, STD	23.26 ± 22.93 a	5.38 ± 6.54 b	0.44 ± 0.53 b	2.11 ± 2.46 b
Nitrogenous compounds							
1-Ethyl-1H-pyrrole	617-92-5	808/806	MS, RI, STD	748.12 ± 1175.84 a	1045.09 ± 332.94 a	64.02 ± 64.02 b	64.49 ± 74.35 b
1-Butyl-1H-pyrrole	589-33-3	937/941	MS, RI	5.4 ± 13.24 ab	11.68 ± 6.23 a	0 ± 0 b	0 ± 0 b
Caffeine	58-08-2	1842/1842	MS, RI	0.22 ± 0.54 a	0 ± 0 a	0 ± 0 a	0 ± 0 a
o-Tolunitrile	529-19-1	1134/1135	MS, RI	210.18 ± 237.22 a	34.17 ± 4.65 b	2.26 ± 0.92 b	9.08 ± 11.2 b
2,4-Dimethyl-3-ethylpyrrole	517-22-6	-/1045	MS, STD	234.35 ± 290.74 a	315.59 ± 131.86 a	14.26 ± 10.24 b	15.35 ± 15.94 b
2-Amino-5-methylbenzoic acid	2941-78-8	-/901	MS	261.44 ± 36.42 a	231.53 ± 263.98 a	68.5 ± 44.28 b	47.11 ± 25.43 b
3,5-Diethyl-2-methyl pyrazine	18138-05-1	1150/1156	MS, RI	24.69 ± 60.16 b	80.73 ± 20.4 a	0.45 ± 0.52 b	1.16 ± 0.63 b
2,3-Diethyl-5-methylpyrazine	18138-04-0	-/1153	MS, STD	6.25 ± 15.19 b	22.31 ± 5.18 a	0.09 ± 0.1 b	0.19 ± 0.16 b
Methyl anthranilate	134-20-3	1336/1337	MS, RI, STD	1015.82 ± 2037.28 ab	1452.42 ± 617.44 a	36.23 ± 33.71 b	65.21 ± 99.75 b
2,5-Dimethyl-3-ethylpyrazine	13360-65-1	1079/1076	MS, RI	76.98 ± 164.5 a	311.45 ± 93.86 b	2.48 ± 2.74 b	4.3 ± 3.51 b
2-Methyl-5-ethylpyrazine	13360-64-0	998/994	MS, RI	301.75 ± 623.85 ab	599.82 ± 340.49 a	14.73 ± 5.12 b	19.63 ± 11.57 b
Indole	120-72-9	1289/1288	MS, RI, STD	239.83 ± 297.89 a	11.05 ± 4.94 b	8.71 ± 8.86 b	1.67 ± 2.12 b
1-Methyl-1H-pyrrole-2-carboxaldehyde	1192-58-1	913/921	MS, RI, STD	1.16 ± 1.02 a	0 ± 0 b	0.24 ± 0.39 b	0.22 ± 0.32 b
Aldehyde compounds							
Citral	5392-40-5	1268/1268	MS, RI	7389.23 ± 8424.4 a	3.94 ± 0.44 b	48.97 ± 69.74 b	81.71 ± 202.4 b
*β*-Homocyclocitral	472-66-2	1254/1255	MS, RI	18.32 ± 21.23 a	0.02 ± 0.03 b	0 ± 0 b	0 ± 0 b
*β*-Cyclocitral	432-25-7	1216/1217	MS, RI, STD	102.02 ± 63.97 a	16.47 ± 7.26 b	3.44 ± 1.94 b	9.66 ± 6.03 b
(*E*,*E*)-2,4-Heptadienal	4313-03-5	1009/1007	MS, RI, STD	170.56 ± 134.43 a	43.77 ± 30.61 b	0.81 ± 1.8 b	32.8 ± 38.91 b
Phenyl acetaldehyde	122-78-1	1040/1039	MS, RI, STD	294.92 ± 264.44 a	74.67 ± 23.37 b	16.46 ± 15.74 b	50.55 ± 103.14 b
Safranal	116-26-7	1196/1196	MS, RI	87.81 ± 88.74 a	6.42 ± 3.98 b	0.87 ± 1.2 b	4.3 ± 5.08 b
Decanal	112-31-2	1203/1203	MS, RI, STD	93.75 ± 48.22 a	9.24 ± 5.22 b	0.39 ± 0.53 b	2.87 ± 2.22 b
Heptanal	111-71-7	899/896	MS, RI, STD	61.56 ± 31.9 a	1.95 ± 1.97 b	0 ± 0 b	4.17 ± 3.54 b
(*Z*)-Citral	106-26-3	1240/1238	MS, RI	7507.57 ± 8322.52 a	3.5 ± 0.94 b	64.23 ± 67.01 b	82.84 ± 186.8 b
Benzaldehyde	100-52-7	957/954	MS, RI, STD	454.84 ± 168.82 a	90.09 ± 11.45 b	27.68 ± 15.45 b	49.95 ± 42.31 b
Hydrocarbon compounds							
2-Methylnaphthalene	91-57-6	1287/1288	MS, RI, STD	8.32 ± 4.51 a	0.64 ± 0.24 b	0.03 ± 0.05 b	0.93 ± 1.61 b
Naphthalene	91-20-3	1178/1177	MS, RI, STD	209.91 ± 181.88 a	11.48 ± 8.91 b	0 ± 0 b	12.47 ± 9.96 b
1-Methylnaphthalene	90-12-0	1302/1304	MS, RI, STD	3.18 ± 1.63 a	0.27 ± 0.11 b	0.02 ± 0.02 b	0.33 ± 0.47 b
Fluorene	86-73-7	1572/1573	MS, RI	0.08 ± 0.14 a	0 ± 0 b	0 ± 0 b	0 ± 0 b
Acenaphthene	83-32-9	/1476	MS	0.22 ± 0.44 a	0 ± 0 b	0 ± 0 b	0.03 ± 0.08 ab
2-Vinyl-naphthalene	827-54-3	1381/1374	MS, RI	1.45 ± 0.88 a	0 ± 0 b	0 ± 0 b	0 ± 0 b
Alloocimene	673-84-7	1128/1127	MS, RI	153.04 ± 112.83 a	23.62 ± 3.2 b	11.79 ± 0.99 b	11.97 ± 2.65 b
Geraniolene	6709-39-3	879/877	MS, RI	11.09 ± 9.92 a	12.99 ± 6.91 a	0.62 ± 0.84 b	0.84 ± 0.76 b
Tridecane	629-50-5	1299/1297	MS, RI	13.31 ± 12.22 a	6.67 ± 5.54 b	1.2 ± 1.39 b	1.89 ± 2.37 b
*D*-Limonene	5989-27-5	1024/1025	MS, RI, STD	258.27 ± 231.56 a	36.42 ± 6.57 b	4.85 ± 7.01 b	7.96 ± 8.09 b
2,7-Dimethyl naphthalene	582-16-1	1415/1412	MS, RI	1.39 ± 0.92 a	0 ± 0 b	0 ± 0 b	0.03 ± 0.09 b
2-Carene	554-61-0	1012/1012	MS, RI	115.94 ± 129.63 a	30.49 ± 6.73 b	12.35 ± 2.02 b	12.23 ± 1.99 b
*β*-Cedrene	546-28-1	1420/1417	MS, RI	166.66 ± 130.48 a	3.77 ± 1.66 b	10.61 ± 13.71 b	31.25 ± 49.55 b
Hexadecane	544-76-3	1599/1596	MS, RI	39.41 ± 35.07 a	3.88 ± 1.26 b	2.24 ± 0.97 b	1.57 ± 0.76 b
meta-Cymene	535-77-3	1024/1020	MS, RI, STD	51.45 ± 50.12 a	7.26 ± 1.73 b	0.61 ± 1.05 b	0.91 ± 1.41 b
*α*-Farnesene	502-61-4	1506/1506	MS, RI	2.8 ± 0.69 a	2.31 ± 0.05 b	2.49 ± 0.51 ab	2.3 ± 0.04 b
*δ*-Cadinene	483-76-1	1521/1522	MS, RI	55.66 ± 55.14 a	0.94 ± 0.55 b	3.79 ± 5.03 b	6.05 ± 3.88 b
Longifolene	475-20-7	1402/1402	MS, RI	150.2 ± 132.38 a	4.36 ± 2.91 b	10.93 ± 8.07 b	31.22 ± 55.36 b
*α*-Ionene	475-03-6	1352/1354	MS, RI	3.69 ± 4.61 a	2.52 ± 1.88 a	0 ± 0 b	0.13 ± 0.23 b
*α*-Cedrene	469-61-4	1409/1409	MS, RI	0.66 ± 1.44 a	0 ± 0 a	0 ± 0 a	0.09 ± 0.25 a
2,6,10-Trimethyltridecane	3891-99-4	1462/1459	MS, RI	5.12 ± 2.18 a	2.25 ± 0.75 b	0.58 ± 0.34 c	0.95 ± 0.57 c
(*E*)-*β*-Ocimene	3779-61-1	1036/1035	MS, RI	1003.92 ± 1027.43 a	112.7 ± 46.49 b	39.78 ± 23.51 b	49.26 ± 28.06 b
1,2-Dihydro-1,1,6-trimethylnaphthalene	30364-38-6	1349/1350	MS, RI	14.96 ± 25.32 a	15.81 ± 6.64 a	0.03 ± 0.03 b	0.44 ± 0.69 b
*γ-*Muurolene	30021-74-0	1484/1471	MS, RI	1.87 ± 2.75 a	0 ± 0 b	0.03 ± 0.07 b	0.02 ± 0.04 b
*α*-Calacorene	21391-99-1	1542/1541	MS, RI	11.41 ± 13.42 a	0.03 ± 0.05 b	0.14 ± 0.32 b	0.22 ± 0.22 b
Pristane	1921-70-6	1706/1702	MS, RI	3.38 ± 3.29 a	0.4 ± 0.54 b	0 ± 0 b	0 ± 0 b
*α*-Ylangene	14912-44-8	1346/1348	MS, RI	4.59 ± 6.03 a	0.01 ± 0.01 b	0.17 ± 0.31 b	0.35 ± 0.32 b
δ-3-Carene	13466-78-9	1011/1017	MS, RI, STD	140.93 ± 109.31 a	66.79 ± 25.09 b	2.46 ± 1.75 c	9.89 ± 4.89 c
Myrcene	123-35-3	991/988	MS, RI, STD	339.1 ± 225.54 a	31.77 ± 4.51 b	12.1 ± 2.95 b	15.92 ± 15.12 b
Toluene	108-88-3	758/751	MS, RI	1163.56 ± 401.83 a	462.85 ± 176.27 b	32.08 ± 8.09 c	229.08 ± 114.89 c
para-Xylene	106-42-3	861/859	MS, RI	514.7 ± 393.68 a	240.56 ± 178.25 b	6.41 ± 4.22 c	54.49 ± 21.34 bc
α-Muurolene	10208-80-7	1500/1498	MS, RI	98.24 ± 107.26 a	3.49 ± 0.78 b	7.54 ± 7.46 b	8.19 ± 3.72 b
Styrene	100-42-5	888/885	MS, RI	147.58 ± 98.38 a	45.48 ± 27.68 b	2.28 ± 1.56 b	25.3 ± 15.74 b
Ketone compounds							
Acetophenone	98-86-2	1062/1063	MS, RI, STD	199.32 ± 155.19 a	36.59 ± 3.09 b	2.23 ± 4.3 b	11.06 ± 7.62 b
*β*-Ionone	79-77-6	1483/1484	MS, RI	267.17 ± 184.23 a	45.6 ± 10.27 b	15.4 ± 2.67 b	21.95 ± 6.9 b
Dl-Camphor	76-22-2	1143/1141	MS, RI, STD	7.28 ± 5.5 a	0.37 ± 0.41 b	0.07 ± 0.15 b	4.26 ± 9.72 b
2,3-Octanedione	585-25-1	982/980	MS, RI	206.87 ± 136.13 a	67.59 ± 38.12 b	13.75 ± 3.26 b	35.11 ± 25.95 b
Hexahydrofarnesyl acetone	502-69-2	1846/1846	MS, RI	0.77 ± 1.89 a	0 ± 0 a	0 ± 0 a	0 ± 0 a
Jasmone	488-10-8	1396/1396	MS, RI, STD	176.27 ± 294.1 a	0.26 ± 0.37 b	1.99 ± 1.59 b	0.56 ± 0.59 b
Geranyl Acetone	3796-70-1	1452/1451	MS, RI, STD	145.64 ± 87.28 a	10.34 ± 1.14 b	2.49 ± 1.06 b	5.8 ± 2.5 b
Dihydro-*β*-ionone	17283-81-7	1438/1436	MS, RI	11.72 ± 1.58 a	10.08 ± 0.04 b	9.78 ± 0.05 b	9.84 ± 0.08 b
Mesityl oxide	141-79-7	775/788	MS, RI	793.17 ± 378.67 a	51 ± 35.76 b	79.28 ± 35.32 b	138.28 ± 126.65 b
L-Menthone	14073-97-3	1154/1144	MS, RI, STD	142.74 ± 68.72 a	6.53 ± 10.09 b	1.52 ± 3.18 b	24.54 ± 22.6 b
*α*-ionone	127-41-3	1425/1425	MS, RI, STD	34.35 ± 28.45 a	15.54 ± 1.9 b	10.4 ± 0.28 b	10.91 ± 0.52 b
Dehydro-*β*-ionone	1203-08-3	1482/1481	MS, RI	21.66 ± 25.06 a	10.74 ± 0.83 ab	9.75 ± 0.05 b	9.79 ± 0.1 b
Methyl heptenone	110-93-0	981/983	MS, RI, STD	161.19 ± 131.58 a	22.09 ± 6.33 b	3.25 ± 2.87 b	13.33 ± 5.92 b
Ester compounds							
Ethyl benzoate	93-89-0	1170/1168	MS, RI, STD	1.11 ± 0.85 a	0.1 ± 0.09 b	0.03 ± 0.02 b	0.05 ± 0.05 b
Methyl benzoate	93-58-3	1091/1091	MS, RI, STD	4.74 ± 4.39 a	2.07 ± 0.96 b	0.11 ± 0.07 b	0.4 ± 0.43 b
Diisobutyl phthalate	84-69-5	1869/1870	MS, RI	14.81 ± 10.96 a	0.8 ± 0.52 b	0.31 ± 0.11 b	0.18 ± 0.07 b
Ethyl Palmitate	628-97-7	1992/1992	MS, RI, STD	0 ± 0 b	0 ± 0 b	0.74 ± 0.9 a	0.44 ± 0.74 ab
(*Z*)-3-Hexen-1-yl isovalerate	35154-45-1	1184/1235	MS, RI, STD	13.78 ± 14.81 a	0.31 ± 0.24 b	1.13 ± 1.79 b	0.98 ± 1.84 b
(*Z*)-3-Hexen-1-yl hexanoate	31501-11-8	1382/1380	MS, RI, STD	70.01 ± 72.93 a	2.23 ± 3.09 b	8.22 ± 14.2 b	1.58 ± 2.98 b
δ-Tetradecalactone	2721-22-4	/	MS	3.68 ± 2.25 a	0.76 ± 0.32 b	0.12 ± 0.05 b	0.14 ± 0.08 b
(*Z*)-3-Hexen-1-yl benzoate	25152-85-6	1566/1568	MS, RI, STD	11.31 ± 9.15 a	1.86 ± 0.79 b	1.51 ± 0.14 b	1.65 ± 0.22 b
(*Z*)-3-Hexen-1-yl butyrate	16491-36-4	1186/1185	MS, RI, STD	17.35 ± 18.12 a	0.2 ± 0.1 b	2.16 ± 3.52 b	0.35 ± 0.45 b
Benzyl acetate	140-11-4	1160/1162	MS, RI, STD	16.96 ± 11.29 a	2.86 ± 0.6 b	0.48 ± 0.31 b	0.98 ± 0.87 b
Ethyl hexanoate	123-66-0	996/997	MS, RI	17380.43 ± 16045.79 a	66.66 ± 13.46 b	1184.3 ± 814.84 b	963.44 ± 761.99 b
Ethyl nonanoate	123-29-5	1294/1295	MS, RI	622.07 ± 347.71 a	7.03 ± 5.22 b	49.43 ± 14.46 b	32.48 ± 41.07 b
Methyl salicylate	119-36-8	1191/1191	MS, RI, STD	158.34 ± 84.82 a	45.37 ± 14.95 b	2.32 ± 1.02 c	4.6 ± 6.96 bc
Linalyl acetate	115-95-7	1257/1226	MS, RI, STD	193.27 ± 272.59 a	11.07 ± 4.4 b	1.2 ± 0.61 b	1.9 ± 1.37 b
Methyl palmitate	112-39-0	1926/1926	MS, RI, STD	0 ± 0 c	8.04 ± 6.78 a	3.98 ± 1 b	3.06 ± 1.78 b
Ethyl octanoate	106-32-1	1196/1196	MS, RI, STD	11.84 ± 9.17 a	0 ± 0 b	0.57 ± 0.17 b	0.57 ± 0.45 b

^a^ Retention index of compounds in reference. ^b^ Retention index of compounds on HP-5MS. ^c^ “MS” mass spetrum comparison using NIST17 library. “RI” retention index in agreement with literature value. “STD” confirmed by authenic standards. ^d^ Different letters indicated significant difference.

## Data Availability

The data are available from the corresponding author.

## Supplementary Materials

The following supporting information can be downloaded at: https://www.mdpi.com/article/10.3390/foods12010027/s1, Supplement Material S1: The detail information of sensory evaluation; Figure S1: Differences of the volatile categories (A) and discriminatory volatiles (B) among the four aroma types of yellow tea; Figure S2: Score plot of OPLS-DA 1 for floral fragrance type versus high-fired fragrance type with R2X (cum): 0.565, R2Y (cum): 0.989, Q2 (cum): 0.984 (A) and permutation testing of the OPLS-DA 1 (B); Score plot of OPLS-DA 2 for floral fragrance type versus fresh fragrance type with R2X (cum): 0.557, R2Y (cum): 0.99, Q2 (cum): 0.985 (C) and permutation testing of the OPLS-DA 2 (D); Score plot of OPLS-DA 3 floral fragrance type versus corn-like fragrance type with R2X (cum): 0.504, R2Y (cum): 0.993, Q2 (cum): 0.989 (E) and Permutation testing of the OPLS-DA 3 (F); Score plot of OPLS-DA 4 for floral fragrance type versus fresh fragrance type with R2X (cum): 0.595, R2Y (cum): 0.989, Q2 (cum): 0.985 (G) and permutation testing of the OPLS-DA 4 (H); Score plot of OPLS-DA 5 for floral fragrance type versus fresh fragrance type with R2X (cum): 0.511, R2Y (cum): 0.994, Q2 (cum): 0.99 (I) and permutation testing of the OPLS-DA 5 (J); Score plot of OPLS-DA 6 for floral fragrance type versus fresh fragrance type with R2X (cum): 0.479, R2Y (cum): 0.991, Q2 (cum): 0.98 (K) and permutation testing of the OPLS-DA 6 (L); Figure S3: Heatmap of the 47 discriminatory aroma compounds in the different aroma types of yellow tea; Table S1: The main volatile compounds in different raw material or processing parameters of yellow tea; Table S2: Yellow tea samples information; Table S3: The standard curve of identified volatile compounds in yellow tea; Table S4: The OAV values of the volatile compounds identified in Yellow tea.
