# Supplementary material for: Characterization of the Key Aroma Compounds in Different Aroma Types of Chinese Yellow Tea
Source: Foods. 2022 Dec 21;12(1):27. doi: 10.3390/foods12010027 (PMC9818532; doi:10.3390/foods12010027)
Supplement: Supplementary file 1 [file foods-12-00027-s001.zip › supplementary file - revised for final 12.03/Figure S2.pdf]

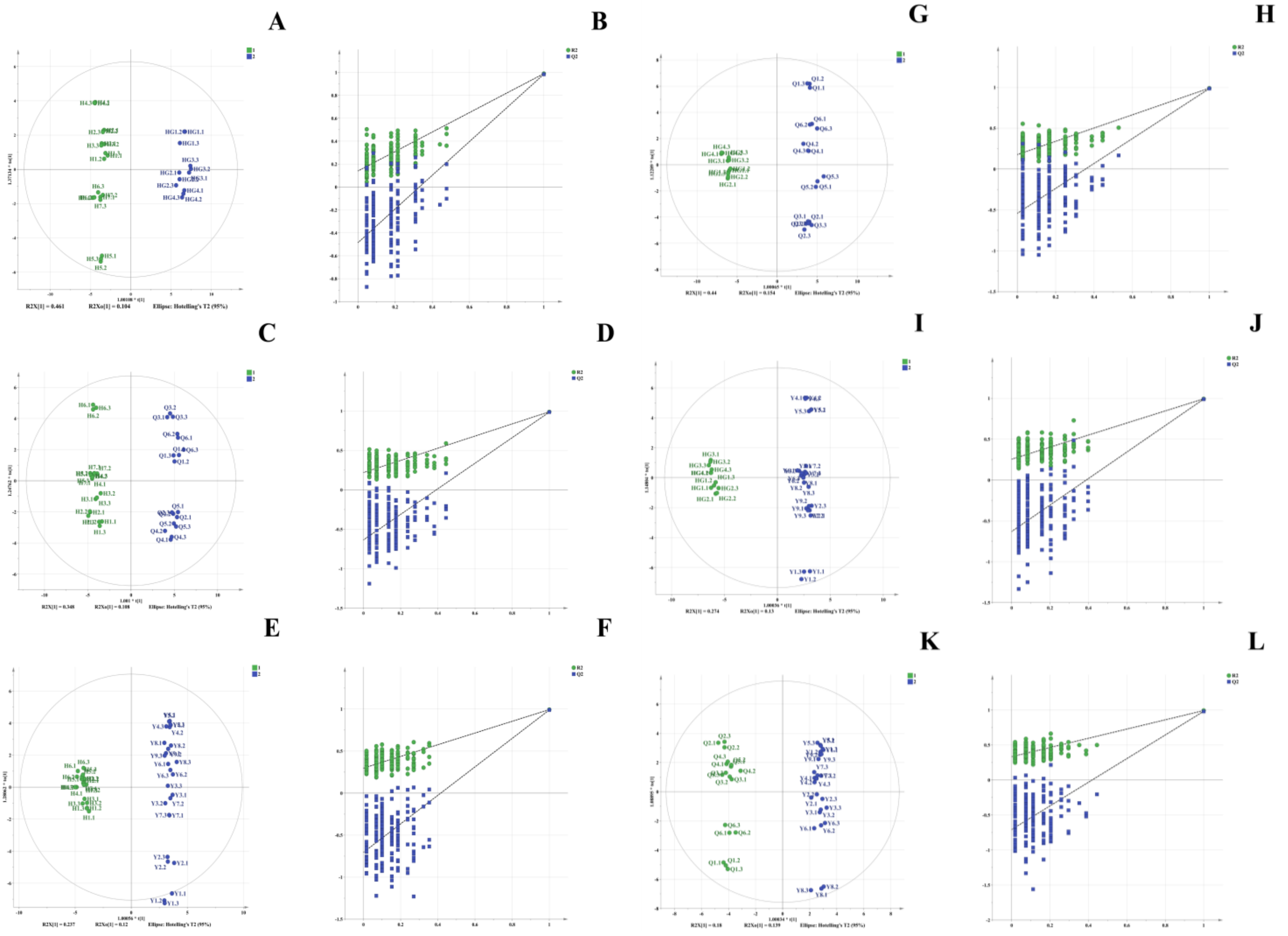


**Figure S2**. Score plot of OPLS-DA 1 for floral fragarance type versus high-fired fragarance type with R2X (cum): 0.565, R2Y (cum): 0.989, Q2 (cum): 0.984 (A) and permutation testing of the OPLS-DA 1 (B); Score plot of OPLS-DA 2 for floral fragarance type versus fresh fragarance type with R2X (cum): 0.557, R2Y (cum): 0.99, Q2 (cum): 0.985 (C) and permutation testing of the OPLS-DA 2 (D); Score plot of OPLS-DA 3 floral fragarance type versus corn-like fragarance type with R2X (cum): 0.504, R2Y (cum): 0.993, Q2 (cum): 0.989 (E) and Permutation testing of the OPLS-DA 3 (F); Score plot of OPLS-DA 4 for floral fragarance type versus fresh fragarance type with R2X (cum): 0.595, R2Y (cum): 0.989, Q2 (cum): 0.985 (G) and permutation testing of the OPLS-DA 4 (H); Score plot of OPLS-DA 5 for floral fragarance type versus fresh fragarance type with R2X (cum): 0.511, R2Y (cum): 0.994, Q2 (cum): 0.99 (I) and permutation testing of the OPLS-DA 5 (J); Score plot of OPLS-DA 6 for floral fragarance type versus fresh fragarance type with R2X (cum): 0.479, R2Y (cum): 0.991, Q2 (cum): 0.98 (K) and permutation testing of the OPLS-DA 6 (L).
