# Supplementary material for: Characterization of the Key Aroma Compounds in Different Aroma Types of Chinese Yellow Tea
Source: Foods. 2022 Dec 21;12(1):27. doi: 10.3390/foods12010027 (PMC9818532; doi:10.3390/foods12010027)
Supplement: Supplementary file 1 [file foods-12-00027-s001.zip › supplementary file - revised for final 12.03/Figure S3.pdf]

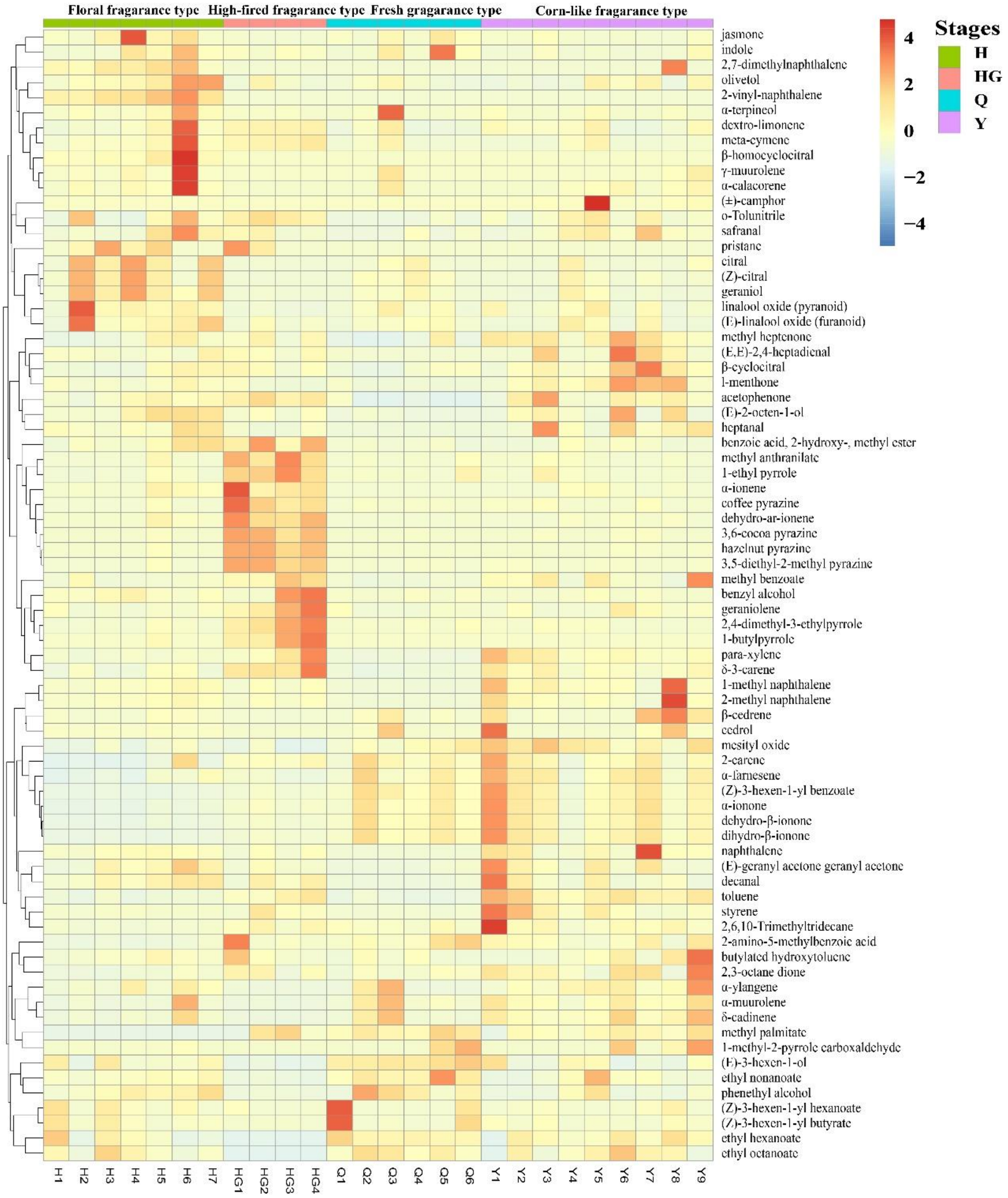

Figure S3. Heatmap of the 47 discriminatory aroma compounds in the different aroma types of yellow tea.
