# Supplementary material for: Characterization of the Key Aroma Compounds in Different Aroma Types of Chinese Yellow Tea
Source: Foods. 2022 Dec 21;12(1):27. doi: 10.3390/foods12010027 (PMC9818532; doi:10.3390/foods12010027)
Supplement: Supplementary file 1 [file foods-12-00027-s001.zip › supplementary file - revised for final 12.03/Supplement material S1.pdf]

Supplement material S1: The detail information of sensory evaluation

The sensory analysis was performed in the tea sensory test room of Hunan Agricultural University. The tea sensory test room was developed according the Chinese standards "General requirement of the tea sensory test room (GB/T18797-2012). Such as, the lighting of the room must ≥750lx, the room temperature 15℃ ~27℃, relative humidity ≤70% and so on. Before the experiment, each panelist had to complete a 60-hours of sensory evaluation training with different aroma types of yellow tea in 20 days. The assessors have been trained to recognize, describe, and quantify the intensity of different aroma character through specific training with yellow tea infusion.

Each yellow tea sample coded with a three-digit random code, and samples were presented to the panelists in random order during each session (10:00 a.m. and 15:00 p.m.), and panelists had at least a 5-minuts break after each sample evaluation to reduce flavor carryover. For the cupping method, the tea infusion preparation followed the standard of the traditional sensory evaluation of yellow tea (GB/T 23776-2018). Briefly, 3.0 g of tea sample was put into a 150-mL special cup (cylindrical cup) and filled with freshly boiling water for 5 min, and the tea infusion was filtered into a special bowl. The tea leaves was stored in the cup and was kept on a temperature-controlled glass plate controlled at 50 °C for aroma evaluation.

During that time, panelists individually evaluated samples and wrote down whatever characteristics they found in each sample. After all samples were evaluated, the panel leader collected all the descriptors and organized the description in the form of a aroma wheel. Then, the panel leader led several discussions to reach agreement on the collected descriptors. Finally, the typical aroma attributes were obtained to describe the overall aroma characteristics of yellow tea. Then, each assessor rated the intensity of the typical aroma attributes by a ten-point scale (0, 5, and 10 represented "not perceptible", "moderate", and "strong", respectively). Each sample was evaluated three times.
