# Supplementary material for: Characterization of the Key Aroma Compounds in Different Aroma Types of Chinese Yellow Tea
Source: Foods. 2022 Dec 21;12(1):27. doi: 10.3390/foods12010027 (PMC9818532; doi:10.3390/foods12010027)
Supplement: Supplementary file 1 [file foods-12-00027-s001.zip › supplementary file - revised for final 12.03/Table S1.pdf]

Table S1: The main volatile compounds in different raw material or processing parameters of yellow tea

Raw material /processing parameters	Types	Compounds	Reference
Different raw material	Bud-leaf yellow tea	benzeneacetaldehyde	Ref 1
	Bud-leaf yellow tea	ethyl hexanoate, benzyl alcohol, geraniol, phenylethyl alcohol, citral, neral, myrcene	
	Multi-leaf yellow tea	1-methylpyrrole-2-carboxaldehyde, 3-ethyl-2,5-dimethylpyrazine, 2-ethyl-5-methylpyrazine, 2,3-diethyl-5-methylpyrazine	
	Mengdinghuangya (bud yellow tea)	2-methyl-butanal, 1-Ethylpyrrole-2-carboxaldehyde, (E)-linalool oxide (furanoid), Methyl salicylate	Ref 2
	Pingyanghuangtang (bud yellow tea)	(Z)-3-hexen-1-ol, styrene, (E)-2-heptenal, Benzaldehyde, 1-octen-3-ol, (E,E)-2,4-heptadienal, (E,E)-3,5-octadien-2-one, (Z)-linalool oxide (furanoid), (E,Z)-3,5-octadien-2-one, Linalool, Naphthalene, Indole, α-ionone, geranyl acetone, trans-β-ionone, dihydroactinidiolide	
	Weishanhuangcha (little yellow tea)	phenylethyl alcohol, geraniol, biphenyl, (Z)-jasmone, coumarin	
Different roasting degree	Small fire (120~130◦C, 30 min)	4-(2,6,6-trimethylcyclohexa-1,3-dienyl)but-3-en-2-one, 2,4,5-trimethyloxazole, trans-β-ocimene, 2-ethyl-3,5-dimethylpyrazine, 2,5-diethylpyrazine, linalool, 2,6-dimethyl-2,4,6-octatriene, 2,3-diethyl-5-methylpyrazine, 3,5-diethyl-2-methylpyrazine, trans-β-ionone	Ref 3
	Medium fire (130~140◦C, 3.5 h)	trans-calamenene, 1-methyl-4-(1-methylethylidene)cyclohexene, 2,4,5-trimethyloxazole, 3-ethyl-2,4-dimethyl-1H-pyrrole, trans-β-ocimene, 2-ethyl-3,5-dimethylpyrazine, linalool, 2,6-dimethyl-2,4,6-octatriene, (E,Z)-2,6-dimethyl-2,4,6-octatriene, 2,3-diethyl-5-methylpyrazine, 3,5-diethyl-2-methylpyrazine, trans-β-ionone, trans-calamenene	
	Old fire (145~155◦C, 3.5 h)	cadala-1(10),3,8-triene, 1,7-dimethyl-naphthalene, 2,4,5-trimethyloxazole, 3-ethyl-2,4-dimethyl-1H-pyrrole, trans-β-ocimene, 2-ethyl-3,5-dimethylpyrazine, 2,5-diethylpyrazine, linalool, 2,6-dimethyl-2,4,6-octatriene, (E,Z)-2,6-dimethyl-2,4,6-octatriene, 2,3-diethyl-5-methylpyrazine, 3,5-diethyl-2-methylpyrazine, trans-β-ionone, trans-calamenene	

1. Jiang R.G.; Huang Y.; Jin Y.L.; Huang F.F.; Liu Z.H.; Huang J.A.; Li Q. Analysis of Characteristic Aroma Components of Different 399 Grades of Yellow Tea. Food Sci. 2021, 42, 89-98.
2. Shi Y.L.;Wang M.Q.;Dong Z.B.;Zhu Y.;Shi J.;Ma W.J.;Lin Z., Lv H.P. Volatile components and key odorants of Chinese yellow tea (Camellia sinensis). LWT-Food Sci Technol. 2021, 146, 111512.
3. Guo X.;Ho C. T.;Schwab W., Wan X. Effect of the roasting degree on flavor quality of large-leaf yellow tea. Food Chem. 2021, 347, 129016.
